# Impact of virtual continued medical education on carbon footprint and awareness of digital sobriety: A retrospective cross-sectional study among public health professionals in India

**DOI:** 10.3389/fpubh.2023.1118685

**Published:** 2023-03-13

**Authors:** Aravind P. Gandhi, Bijaya Kumar Padhi, Kapil Goel, Amarjeet Singh, Om Prakash Kansal, Tareq Al-Ahdal

**Affiliations:** ^1^Department of Community Medicine, ESIC Medical College and Hospital, Hyderabad, India; ^2^Department of Community Medicine & School of Public Health, PGIMER, Chandigarh, India; ^3^Global Health, Becton Dickinson, New Delhi, India; ^4^Institute of Global Health (HIGH), Heidelberg University, Heidelberg, Germany

**Keywords:** environment, carbon footprint, India, telemedicine, virtual education

## Abstract

**Background:**

Due to the COVID-19 pandemic, physical meetings and continuing medical education (CMEs) are being conducted in virtual mode. Digital sobriety has been advocated as a strategy for controlling the environmental emission from online events. The present study was undertaken to assess the impact of virtual CMEs on the environment and the participants' perception, knowledge, attitude, and practices of digital sobriety during the CMEs.

**Methods:**

A retrospective cross-sectional Google form-based online study was conducted among the 1,311 registrants of 23 virtual CMEs hosted in India. A pre-tested English questionnaire was used to collect the data. The potential carbon footprint of the significant physical CME activities and the carbon emission (CE) of the virtual CMEs were estimated. Among the registrants contacted, 251 consented and participated in the study.

**Results:**

The CE of the virtual CMEs was 0.787 metric tons of carbon dioxide equivalent (MT CO_2_ Eq). If the CMEs were conducted physically, the potential CE was estimated to be 290.094 MT CO_2_ Eq. The awareness rate of digital sobriety was 35%. Most of the participants (58.7%) from the current study preferred the hybrid mode of CMEs.

**Conclusions:**

Virtual, digitally sober CMEs have reduced the potential CE by 99.7% compared to physical CMEs in India. The awareness and knowledge about digital sobriety is low in India. Knowledge, networking, social interactions, and overall satisfaction were relatively lower in the virtual mode of CMEs than in the physical mode.

## Introduction

Climate change is real, and every sector needs to do its part to mitigate and prevent the phenomenon. The environment is an integral part of human health and it should be considered and protected in all health-related activities. Continued Medical Education (CME) is essential to medical education and practices. The regulatory and supervisory bodies for medicine require mandatory CME credit hours from physicians to renew their practice licenses (1). Owing to the COVID-19 pandemic, meetings and teachings are being conducted in virtual mode. Telemedicine has been explored extensively in all aspects of medicine. In this pandemic, CMEs are also being conducted in virtual mode as a form of telemedicine. The environmental impact of this switch is mainly positive (2, 3). However, the online events have certain ecological consequences as well. A sizable carbon emission is contributed by servers, data consumption, software, and network usage (4). Digital and information technologies are credited with 3.7% of all carbon emissions (CE) (5). A single email generates 4 g of CO_2_ equivalent. Similarly, every digital activity, such as streaming a video, visiting a webpage, or downloading a file, has its own carbon emission. Hence, strategies must be adopted to reduce carbon emission from virtual events. Digital sobriety has been advocated as a strategy for controlling the environmental emission from online events and enhance sustainable consumption of digital technology. Digital sobriety mainly consists of “buying the least powerful equipment possible, changing them the least often possible, and reducing unnecessary energy-intensive uses” (5). Institutions and individuals can adopt the digital sobriety strategies. Although the existing literature has quantified the emission from virtual events (6), including digitally sober scenarios (4, 7), they did not incorporate the duration spent in the virtual devises and mean years of usage of the devices, which are essential to improve the accuracy of the CE estimate. Also, no studies on the awareness and practice of digitally sober measures among attendees of virtual event could be found. Additionally, the impact of such a virtual switch of the CMEs on other essential domains of learners, such as knowledge, attitude, social relationships, and environmental effects from the delegates' perspective must also be studied in Indian settings. Therefore, the current study was conducted to assess the impact of virtual CMEs on the environment and participants' perception, knowledge, attitude and practices of digital sobriety in India, 2021.

## Materials and methods

### Study design

A Retrospective cross-sectional Google form-based online study.

### Study population

Registrants of the Virtual Preconference CMEs conducted during the 48th “Annual Conference of the Indian Association of Preventive and Social Medicine (IAPSMCON 2021)”-−1,311. Registrants who did not give their consent were excluded.

### Study settings

The IAPSMCON 2021 was held from 19th to 21st March 2021, which was the first in its series to be held virtually, due to the COVID-19 scenario. The Department of Community Medicine and School of Public Health, Postgraduate Institute of Medical Education and Research (PGIMER) at Chandigarh, India, organized the conference. There were 2,046 registrants for the conference. The most recent annual conference of the association (IAPSMCON 2023) was conducted offline with 1,300 participants. A series of 23 CMEs was conducted before the conference from 7th to 18th March 2021 by the organizers of the IAPSMCON 2021. Due to the COVID-19 situation, all CMEs were hosted in virtual mode by the Institute located in Chandigarh. There were 1,311 participants registered for the CMEs. The CMEs were conducted virtually through Zoom meetings. The registered participants were mailed the Zoom meeting code and password to attend the CME by the respective convener of the CME. The duration of the CMEs ranged from 2 to 11 hours.

### Sample size and sampling technique

A complete enumeration of all registered participants was done-−1,311.

### Data collection

The email IDs of the CME registrants were obtained from the IAPSMCON 2021 preconference CME database. A pre-tested, structured English questionnaire was sent as a Google form to the participants after all the CMEs were over. A maximum of three reminders were sent to registrants. For the percentage of delegates who would have attended the physical conference, the potential carbon footprint of the major physical conference activities, such as travel, lodging, meals, and certificates, was computed by extrapolating the responses given by the study participants. Similarly, the CE of the virtual CMEs was estimated.

### Estimation of the CEs: Physical mode

The potential CE from the travel-related activities of the CME participants was estimated based on the mode of travel, which the probable attendants would have preferred to reach the CME venue, had it been in physical mode. The potential distance traveled (distance from the state capital of the participants to Chandigarh) (8, 9), and the CE per kilometer for each mode of travel (air, rail, and road) were used to calculate the CE for the transport activities (10–12). Based on an online tool (13), the potential CE from the accommodation of the CME participants and speakers and the CME halls were estimated. Three meals and two snack course was formulated, and the CE was estimated by tool which is available online (My Emissions) (14).

### Estimation of the CEs: Virtual mode

The CE of the virtual platform, Zoom (https://zoom.us/), used to conduct the CMEs was assessed based on the number of users, usage hours and server usage.

The CE of the server were estimated by [1] (6):


(1) $$\begin{array}{l} {\text{E}}_{\text{e}}^{*}{\text{S}}^{*}\text{Ws}*{\text{H}}_{\text{c}} \end{array}$$

Where Ee denotes the electricity emissions (kg CO_2_-eq/kWh), S denotes number of servers (which was taken as 1), Ws represents the server's power rating (kW/server), and Hc is the total time for which the CMEs happened. CE from other virtual resources such as emails sent (15), webpages loaded (16), and devise usage to attend the CMEs (6), by the participants were also estimated.

The detailed methodology applied for estimating the CEs of the virtual and potential physical CMEs is mentioned in Supplementary material 1. The frequency of the potential carbon emission saved was calculated for each activity, and proportions were calculated to assess the contribution from each activity to total emissions.

A pre-tested, semi-structured questionnaire was applied to determine the knowledge, attitude and practices of digital sobriety among the participants. The items in the questionnaire assessed their awareness of the concept, activities which lead to carbon emission, their perception toward environmental protection by the virtual meeting, and their actual conduct and activities in the virtual systems during the CMEs.

### Data analysis

MS Excel and SPSS v20 were used for data collation and analysis. In both the physical and online versions of the conferences, the potential CE of different activities was estimated. The total CE between the two versions of the conference was compared. The respondents' knowledge, perception and satisfaction levels from the virtual CME were expressed in proportions. A score was formulated to assess awareness and practice of digital sobriety. Continuous variables were assessed for normality (Kolmogorov-Smirnov test). Chi-square and Fisher's exact test were applied for testing the significance between categorical variables. Age, Gender, official designation of the participant, experience duration, duration of CME attended were the variables considered in the univariate model. The Mann-Whitney test was applied to assess the significance in the difference in continuous data between the respondents who were aware and not aware of digital sobriety. A multivariate analysis by multiple logistic regression was planned by including the variables which had a *p* < 0.1 in the univariate analysis.

### Ethics

Ethical approval was taken from the Institutional Ethics Committee, PGIMER, Chandigarh. Since the study was conducted through google forms, virtual informed consent was obtained from the participants before starting the questionnaire providing all relevant information. All data collected was kept confidential and secure.

## Results

Among the contacted registrants, 251 consented and completed the study (response rate: 19%). The median age of the participants was 29 years and the majority were females (63.7%) and junior residents (55%). Among the respondents, the majority (87.3%) responded that they would have attended the CMEs if it had happened in person mode. Among the respondents, 82.1% (206) participated in the CMEs for a total median duration of 4 hours. The socio-demographic characteristics of the respondents are enumerated in Table 1.

**Table 1:** Socio-demographic characteristics of the respondents (*N* = 251).

**Variable**	**Frequency**	**Percentage**
Age median (IQR)	29 (27, 36) years	
**Gender**		
Male	90	35.9
Female	160	63.7
Prefer not to say	1	0.4
**Designation**		
Junior resident	138	55
Senior resident	19	7.6
Public health professional	24	9.6
PhD/Researcher	15	6.0
Faculty	46	18.3
Others	9	3.6
Years of work in the field Median (IQR)	2 (1.5, 5) years	
**Would have attended physical CME**		
Yes	209	87.3
No	42	16.7
**Preferred mode of transport for physical CME (*****N*** = **209)**		
Airways	103	49.3
Railways	62	29.7
Roadways	44	21.1
**Preferred mode of the CME**		
Physical	54	26.2
Virtual	24	11.7
Hybrid	121	58.7
Anything (no preference)	7	3.4
**Attended CMEs during IAPSMCON 2021**		
Yes	206	82.1
No	45	17.9
Total attended CME duration [median (IQR)]	4 (3, 5) hours	

As per the responses given by the study participants, the carbon emission due to the virtual CMEs was evaluated to be 0.787 metric tons carbon dioxide equivalent (MT CO_2_ Eq) (Supplementary material 2). During the CMEs, most of the CE was from streaming the CME in the Zoom platform (75.2%), followed by device usage (20.7%) (Table 2).

**Table 2:** Carbon emission under various aspects during the virtual CMEs.

**Emission heads**	**MT CO_2_ Eq**
Video streaming	0.592
Emails	0.015
E certificates	0.003
Website page views	0.012
Device usage	0.163
Zoom server usage	0.002
**Total**	**0.787**
**Per capita emission**	**0.001**

Among the respondents, 87% committed that they would have attended the CMEs if it had been in physical mode. The potential CE of the probable 1,141 attendees (87% of the registrants), if the CMEs were held physically, was estimated to be 290.094 MT CO_2_ Eq (Table 3; Supplementary material 3, 4). The potential per capita emission of the physical CMEs was 0.254 MT CO_2_ Eq. The transportation activity that the delegates would have engaged in to attend the CMEs would have produced the majority of the potential CE from the physical-mode CMEs (74.8%). This was followed by accommodation (19.8%) and food (5.3%). Overall, the CE prevented in the CMEs by choosing the online mode was 289.308 MT CO_2_ Eq (99.7%).

**Table 3:** Potential CE under various aspects during the physical CMEs.

**Emission heads**	**Physical mode (CO_2_ Eq MT)**
Transport	217.030
Accommodation	57.570
Certificates	0.007
Food	15.488
**Total**	290.095
**Per capital emission**	**0.254**

Among the CME attendees in our study (206), the awareness rate of “digital sobriety” was 35% (72). Majority of the attendees who responded (59.7%) did not know that the most commonly done day to day digital activities such as sending an email, watching a video, downloading a PDF file, opening a web page, sending a video through internet emits carbon (Table 4).

**Table 4:** Knowledge, attitude and practices of digital sobriety among the participants (*N* = 206).

**Variables**	**Frequency**	**Percentage**
**Knowledge**		
Aware of the term “digital sobriety”		
Yes	72	35.0
No	134	65.0
Digital activities that cause carbon emission		
Sending an email	23	12.1
Watching a 10 mins video in HD	60	29.1
Doing a google search	26	12.6
Opening a web page	22	10.7
Sending video file through internet	37	18
Downloading a PDF file	20	9.7
None of the above	123	59.7
**Attitude**		
Do not cause ANY adverse environment impact when they attend the virtual CMEs		
Strongly disagree	64	31.1
Disagree	52	25.2
Neither disagree nor agree	47	22.8
Agree	24	11.7
Strongly agree	19	9.2
Virtual mode of CMEs reduces the adverse environmental impact caused by		
the physical mode		
Strongly disagree	45	21.8
Disagree	55	26.7
Neither disagree nor agree	50	24.3
Agree	31	15.0
Strongly agree	25	12.1
Attending virtual CMEs is a contribution to environment protection		
Yes	188	91.3
No	18	8.7
**Practices**		
Self-video during virtual CME		
Video camera was switched off all the time	104	50.5
Video camera was switched OFF most the time (more than 50% of the meeting duration)	75	36.4
Video camera was switched on all the time	10	4.9
Video camera was switched ON most of the time (more than 50% of the meeting duration)	17	8.3
Time of switching on the camera during the CMEs		
All the time	7	3.4
Most of the time (>50% of the duration)	15	7.3
Never during the meeting	85	41.3
When they need to speak or ask question	99	48.1

Most of the participants had a positive attitude towards virtual CMEs that they contribute to environmental protection by attending such virtual CMEs (91.3%).

Regarding the practices of digital sobriety acts, most had switched off their video cameras at all (50.5%) or most of the time (36.4%) and turned them on only when they needed to speak or ask a question (48.1%).

Among the participants in our study, the awareness rate of digital sobriety was 35%. No significant association was found between the respondents' socio-demographic factors and their awareness of digital sobriety. Similarly, the two groups' total knowledge attitude and practice scores estimated toward the environmental aspects of the virtual CME were also similar (Table 5).

**Table 5:** Association between the awareness of digital sobriety and the socio-demographic, KAP domains of environmental aspects of virtual CME.

**Variable**	**Aware about the term “Digital Sobriety”**				***p* value**
	**Yes**		**No**		
	***n***	**%**	***n***	**%**	
**Gender**					
Male	26	36.6	45	63.4	0.744^a^
Female	46	34.3	88	65.7	
**Designation**					
Junior resident	39	34.2	75	65.8	0.932^b^
Senior resident	7	43.8	9	56.3	
Public health professional	7	41.2	10	58.8	
PhD/Researcher	4	30.8	9	69.2	
Faculty	14	34.1	27	65.9	
Others	1	20	4	80	
Age, median (IQR)	29.5 (28, 35.8)	–	29 (27, 35)	–	0.632^c^
Years of work in the field, median (IQR)	8 (4, 12)	–	8 (4, 16)	–	0.315^c^
Total duration of CME attended, median (IQR)	4 (3, 4)	–	4 (3, 6)	–	0.235^c^
Total KAP score	9 (7, 11)	–	8 (6, 11)	–	0.224^c^

^a^Chi-square test.

^b^Fishers exact test.

^c^Mann-Whitney Test.

The majority of CME attendees expressed that knowledge acquired (49.5%), interactive sessions (59.2%), networking achieved with peers (72.8%), social relationships developed (82%), the ambience (62.1%) and overall satisfaction (54.9%) from the virtual mode of the CMEs have been lower in comparison with that of the physical mode (Table 6).

**Table 6:** Opinion toward the virtual CME in comparison to the physical CMEs among the participants.

**Knowledge acquired in virtual CME**	**Frequency**	**Percentage**
Less than that of physical mode	102	49.5
Same as that of physical mode	65	31.6
More than that of physical mode	39	18.9
**Interactive sessions during the CME**		
Less than that of physical mode	122	59.2
Same as that of physical mode	43	20.9
More than that of physical mode	41	19.9
**Networking achieved with peers**		
Less than that of physical mode	150	72.8
Same as that of physical mode	24	11.7
More than that of physical mode	32	15.5
**Social relationships developed/maintained**		
Less than that of physical mode	169	82.0
Same as that of physical mode	19	9.2
More than that of physical mode	18	8.7
**Ambience (atmosphere of the CME in the virtual platform)**		
Less than that of physical mode	128	62.1
Same as that of physical mode	43	20.9
More than that of physical mode	35	17.0
**Overall satisfaction level with the virtual CME**		
Less than that of physical mode	113	54.9
Same as that of physical mode	61	29.6
More than that of physical mode	32	15.5

## Discussion

Moving academic meetings and conferences to an online platform have been proposed as an effective strategy to reduce the environmental impact of such sessions (20, 21). In the present study, the estimated per capita CE for attending virtual CMEs was 1 Kg of CO_2_ Eq (0.001 MT CO_2_ Eq), while the physical mode of CMEs would have caused a potential per capita CE of 254 Kg of CO_2_ Eq. In comparison, the CE per capita of the people living in India is 2.7 MT CO_2_ Eq (22). Potential CE saved during the CMEs included in the present study by adopting the virtual mode was 289.308 MT CO_2_ Eq (99.7%). This is significant considering the reports that put the estimate of CO_2_ emission by the event industry across the world to be around 10% of global CO_2_ emission (23).

Studies have reported a similar reduction in CE by adopting the virtual mode of academic events over the physical mode. A reduction in CE by 66–200 times has been reported (6, 24). A comparison between the virtual meeting of “American Association for Pediatric Ophthalmology and Strabismus (AAPOS)” in 2021 and the physical version of the meeting which happened in 2022 revealed a reduction of CE between 880 and 1,282 MTCO_2_ due to the virtual version (25). Travel component in physical conferences and meetings is considered a major contributor to carbon emission (26). The potential CE of transport involved in attending the CMEs and returning has been estimated to be the single most important factor in the overall potential of CE. This is in line with previous literature, which also found transport to be the predominant factor (21, 27, 28). Travel-related emissions incurred to present a paper at conferences were pegged at 0.8 MT CO_2_ Eq, worldwide (29).

Although there was a huge reduction in CE owing to the virtual mode of CMEs, the present study delved micro level to assess the digital sobriety among the attendee. The present study found a 35% awareness rate about “digital sobriety” among the attendees. No significant association was found between the socio-demographic factors and their awareness of digital sobriety.

Digital sobriety assumes significance in the context that the carbon footprint attributed to digital activities increases at a rate of 8% annually (17). Most participants had a positive attitude toward virtual CMEs that they contribute to environmental protection by attending such virtual CMEs (91.3%). At the individual level, most of the study participants had switched off their video cameras all (50.5%) or most of the time (36.4%) and turned them on only when they needed to speak or ask a question (48.1%). This assumes importance, since keeping the self-videos switched off by the attendees during the virtual events reduced carbon emissions by about 96% compared to keeping the video switched on all the time (6). Though not all adopted this measure of digital sobriety, the majority reported following it, making the CMEs a digitally sober event. This practice affected the CE contributed by the virtual CME.

Regarding knowledge about digital sobriety, the majority of study participants (59.7%) did not know that the most commonly done day-to-day digital activities, such as sending an email, watching a video, downloading a PDF file, opening a web page, sending a video file through the internet, emits carbon. This is a point of concern, as it denotes inadequate awareness of the environmental impact of digital activities, even among the educated lot in the health sector. Previous studies also showed a lack of awareness among users regarding the environmental impact of the internet and digital products (30, 31). Gnanasekaran et al., in their study, reported poor awareness of the digital carbon footprint of online applications and services used by people who are avid users of such services (31). The role of the internet in CE seems to be underestimated by the users, while estimates report that the internet consumes 10% of the world's electricity (32). The potential reasons behind the underestimate could be a lack of knowledge as per the present study and a previous study (30). Other possible reasons might be accepting internet pollution as a side effect of other benefits from it and unwillingness to act (30). The facilitators and barriers for adopting and practicing eco-friendly online practices at the individual level among participants in our study must be explored through a qualitative study. This is essential since the proportionate share of the internet in pollution has been increasing rapidly over the past decade. Digital devices and support systems emit 3.7% of total greenhouse gas emissions, which is on the rise (32).

The majority of CME attendees expressed that knowledge acquired (49.5%), interactive sessions (59.2%), networking achieved with peers (72.8%), social relationships developed (82%), the ambience (62.1%) and overall satisfaction (54.9%) from the virtual mode of the CMEs have been lower in comparison with that of the physical mode. This indicates the need to understand the determinants for such a relatively lower satisfaction in the virtual mode of the event than in the physical mode, among the study participants. Of their Korean participants in a virtual conference, Kim et al. reported an overall median satisfaction and social exchanges score of 3 and 2.7, respectively, on a 5-point Likert scale (33). Though the overall perception from the Korean study was positive, the methodology differed from the present study.

Although the majority of participants from the current study preferred the hybrid mode of CMEs (physical and virtual modes) followed by the physical mode (26.2%), Kim et al. reported that 50% of their participants preferred the virtual mode of conferences, while 33% had a preference for the conventional physical mode (33).

Lower satisfaction with social relationships and exchanges has been reported in our study, which is in line with previous studies (33, 34). A blended or hybrid mode of academic meetings is suggested to address this limitation of the total virtual mode (25, 35, 36). Since most of the present study participants also preferred a hybrid mode for the CMEs, this can be implemented in our settings in the near future to study the impact on the social relationships and interactions of the participants. Multiple formats within the blended model have been proposed that can be tailored and tested in current settings (18, 19).

According to our literature search, this is the first study from India to report on the awareness and practices of digital sobriety among the attendees of virtual events. The accuracy of the CE of the virtual event was improved by including the details on the video mode, type of device used to attend the event, years of durability and daily hours of usage of the device. However, our findings should be interpreted cautiously due to the following limitations. A low response rate of 19% was found in the study, which could have led to selection bias. Participants attended the CMEs under a single institute and domain (public health), thus limiting its external validity. Potential determinants of the lack of awareness of the environmental impact of the internet and digital activities were not elicited. Future studies should be conducted to explore the determinants of low awareness and knowledge of digital sobriety.

## Conclusions

Virtual, digitally sober CMEs have reduced the potential CE by 99.7% compared to physical CMEs in India. The awareness and knowledge about digital sobriety may be low in India. Knowledge, networking, social interactions, and overall satisfaction were relatively lower in the virtual mode of CMEs than in the physical mode. Participants prefer the hybrid mode of CMEs over exclusive physical or virtual modes. Institutions and individuals must be encouraged to adopt digitally sober online and virtual world strategies, thus promoting sustainable consumption in virtual events.

## Data availability statement

The raw data supporting the conclusions of this article will be made available by the authors, without undue reservation.

## Ethics statement

The studies involving human participants were reviewed and approved by Institute Ethics Committee, PGIMER, Chandigarh, India. The patients/participants provided their Virtual informed consent to participate in this study.

## Author contributions

Conceptualization: AG and KG. Methodology: TA-A, AG, KG, BP, AS, and OK. Formal analysis and investigation: AG, KG, and BP. Writing—original draft preparation: AG. Writing—review and editing: TA-A, KG, BP, AS, and OK. Supervision: KG. All authors read and approved the final manuscript.
