# Supplementary material for: Impact of virtual continued medical education on carbon footprint and awareness of digital sobriety: A retrospective cross-sectional study among public health professionals in India
Source: Front Public Health. 2023 Mar 13;11:1118685. doi: 10.3389/fpubh.2023.1118685 (PMC10040747; doi:10.3389/fpubh.2023.1118685)
Supplement: Supplementary file 1 [file Data_Sheet_1.PDF]

Online Resource 1: Methodology for estimating CEs from virtual and physical modes

Physical Mode

Transportation:

The CO_2 Eq, which would have been emitted due to the travelling by participants for the physical CMEs, was estimated based on the proportion of virtual attendees who would have attended the physical CMEs. Among the 1311 persons registered for CMEs, 87% (1141) would have also participated in the CMEs had it been physical. The potential distance which the attendees had to travel for attending the CMEs was calculated as the shortest distance between the capital city of the state/district and Chandigarh.[1, 2] Then, the two-way distance was calculated. We considered three modes of transportation for travel by the attendees: Rail, air and road. The mode of transport was decided based on the state and country of origin. For the delegates who hail from states other than Punjab, Haryana and Himachal Pradesh, the mode of transport was distributed based on the preference given by the attendees for a potential physical CME. Among them, 49% would have preferred airways as a mode of transport and 30% preferred railways, while 21% preferred roadways.

The capital city of the states of Punjab and Haryana is Chandigarh. The shortest distance between Shimla (capital of Himachal Pradesh) and Chandigarh was less than 120 Kms. Hence, we calculated the distance between Chandigarh and the district headquarters of the attendees from these three states and considered roadways as the mode of transport. A sedan car was assumed to be the vehicle of choice for road travel. Participants from Chandigarh were excluded from the transport analysis. CEs per km for these modes and types of transport were obtained for India.[3–5].

Accommodation & the CMEs hall:

The speakers would have been lodged in a three-star hotel under single occupancy. The attendees were assumed to have taken a shared occupancy (2 per room) in a two-star hotel. The space necessary to conduct the 23 CMEs was calculated under a 4-star hotel. The CE based on the above assumptions was calculated for the hotel rooms as well as the supposed CMEs hall, using an online tool.[6].

Food:

CE from the food served at the CMEs was calculated by formulating a three meals and two snacks. Menu for each meal and snacks were formulated, and the CE of each meal was calculated using an online tool.[7] The online tool used in the present study was 'My Emissions'.

Certificates:

The certificates for the speakers and probable attendees at the CMEs were included to calculate CEs.

Virtual Mode

Virtual platform:

The CMEs were conducted in the Zoom platform with speakers, organizers and the attendees in the platform. Hence, we calculated the CE of the IT platform used in the virtual CMEs under the following heads: Zoom meeting usage by the speakers, organizers and the attendees, server usage at the Zoom data centre, Emails sent to the speakers and registrants, Page views of the website hosting the CMEs, devises used by the attendees for the virtual CMEs. The CE for visiting the CMEs website page was calculated by assuming that each delegate would have visited CME website two time for checking updates. [8] The CE of the server were calculated using the formula [1],[9]

$E_e*S*Ws*H_c$ ----------------------- (1)

Where Ee is the electricity emissions (kg CO_2-eq/kWh), S is the number of servers (assumed to be one), Ws is the power rating of servers (kW/server), and Hc is the total CMEs duration (hour)

The average CE of electricity in India was obtained from the central audit report of the Power ministry.[10] Although Zoom servers are located in India, the CE data for the same could not be found from the literature search. Hence we used the data available from the USA.[9] The devices used for attending the CMEs was obtained from the google form based data collected in the study. Among the ones responded, 62% of the participants used laptop and 32% used smartphones to attend the CMEs. Hence these two devices were taken to estimate the device CO_2 emissions for analysis. The average of the CE emission reported by the laptop brands used by the delegates for attending the CMEs was calculated, and the total CE emission from the laptops was assessed.

Among the smartphones, only one of the brands published the CE per smartphone by Life cycle analysis (LCA). Hence, the data was used for other smartphones as well. Since we did not have data on the models of that brand used by the attendees, we took the average CE of India's top three selling models of that brand.[11, 12] The CE of these devices were obtained based on their LCAs.[9] The average lifetime of the laptops was reported as 3.3 years and mobile phones was reported as 3 years by the respective users in the present study. Average usage of laptops and smartphones per day were reported to be 5 hours and 6 hours, respectively. The median duration of the CMEs attended by the respondents was found to be 4 hours. The CE share of those devices during the CMEs period was calculated based on the formula(2).[9]

$P_c*E_c*Hc/(Y*365.25*H_d)$ ----------------------------- (2)

where Pc is the number of participants which equals the number of devices used (laptop or smartphone), Ec is the devise emissions in kg CO2-eq/devise), Hc is the median duration of the CMEs attended by a delegate in hours, Y is the years of the useful life of the laptop/smartphone, Hd is the daily hours of laptop/smartphone use.
CE happened due to the virtual systems and emails sent to speakers and registrants ,[13] e-certificates generated and sent, virtually, were also estimated.

References:

[1] Flight Distance and Duration Calculator - Airport and Aviation Database - Great Circle Mapper, https://www.greatcirclemapper.net/ (accessed 2 May 2021).

[2] Google Maps, https://www.google.co.in/maps/dir///@30.5697679,76.4819447,10z/data=!4m2!4m1!3e0 (accessed 2 May 2021).

[3] Gajjar C, Sheikh A, Program I. *India Specific Road Transport Emission Factors*. 2015. Epub ahead of print 1 January 2015. DOI: 10.13140/RG.2.2.28564.32646.

[4] Gajjar C, Sheikh A, Program I. *India Specific Rail Transport Emission Factors for Passenger Travel and Material Transport*. 2015. Epub ahead of print 1 January 2015. DOI: 10.13140/RG.2.2.25208.88328.

[5] India GHG Program. *India Specific Air Transport Emission Factors for Passenger Travel and Material Transport For Stakeholder Consultation*. 2015.

[6] Hotel Footprinting Tool, https://www.hotelfootprints.org/ (accessed 22 March 2021).

[7] Food carbon footprint calculator - My Emissions, https://myemissions.green/food-carbon-footprint-calculator/ (accessed 17 May 2021).

[8] What's the Carbon Footprint of Your Website? | Climate Protection | RESET.org, https://en.reset.org/blog/whats-carbon-footprint-your-website-01162020 (accessed 8

May 2021).

[9] Faber G. A framework to estimate emissions from virtual conferences. *Int J Environ Stud*. Epub ahead of print 2021. DOI: 10.1080/00207233.2020.1864190.

[10] Bhawan S, Puram RK. *CO2 Baseline Database for the Indian Power Sector User Guide Government of India Ministry of Power Central Electricity Authority*. 2018.

[11] The most popular iPhones - 2020, https://deviceatlas.com/blog/most-popular-iphones (accessed 17 May 2021).

[12] Environment - Apple, https://www.apple.com/environment/ (accessed 17 May 2021).

[13] Infographic: The Carbon Footprint of the Internet | ClimateCare, https://www.climatecare.org/resources/news/infographic-carbon-footprint-internet/ (accessed 9 April 2021).
